# Canadian Contributions in Fibroblast Biology

**DOI:** 10.3390/cells11152272

**Published:** 2022-07-23

**Authors:** Danah S. Al-Hattab, Sikta Chattopadhyaya, Michael P. Czubryt

**Affiliations:** 1 Institute of Cardiovascular Sciences, St. Boniface Hospital Albrechtsen Research Centre, Winnipeg, MB R2H 2A6, Canada; dalhattab@sbrc.ca (D.S.A.-H.); schattopadhyaya@sbrc.ca (S.C.); 2 Department of Physiology and Pathophysiology, Rady Faculty of Health Sciences, University of Manitoba, Winnipeg, MB R3E 0J9, Canada

**Keywords:** fibroblast, myofibroblast, fibrosis, Canada, cell biology, metabolism

## Abstract

Fibroblasts are stromal cells found in virtually every tissue and organ of the body. For many years, these cells were often considered to be secondary in functional importance to parenchymal cells. Over the past 2 decades, focused research into the roles of fibroblasts has revealed important roles for these cells in the homeostasis of healthy tissue, and has demonstrated that activation of fibroblasts to myofibroblasts is a key step in disease initiation and progression in many tissues, with fibrosis now recognized as not only an outcome of disease, but also a central contributor to tissue dysfunction, particularly in the heart and lungs. With a growing understanding of both fibroblast and myofibroblast heterogeneity, and the deciphering of the humoral and mechanical cues that impact the phenotype of these cells, fibroblast biology is rapidly becoming a major focus in biomedical research. In this review, we provide an overview of fibroblast and myofibroblast biology, particularly in the heart, and including a discussion of pathophysiological processes such as fibrosis and scarring. We then discuss the central role of Canadian researchers in moving this field forwards, particularly in cardiac fibrosis, and highlight some of the major contributions of these individuals to our understanding of fibroblast and myofibroblast biology in health and disease.

## 1. Fibroblasts, Myofibroblasts and Fibrosis

### 1.1. Fibroblast Plasticity

**Figure 1:**
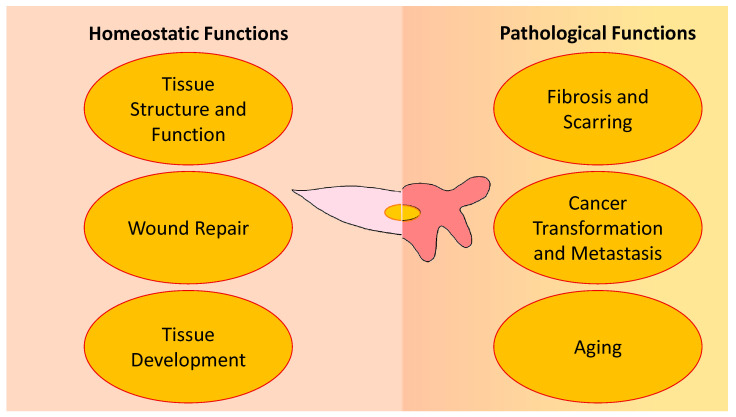
Homeostatic and pathological functions of fibroblasts (**left**) and myofibroblasts (**right**). Fibroblasts normally perform a number of key supportive roles in healthy tissues, and ideally contribute to the non-pathological healing of wounds. In response to physical and chemical signals, fibroblasts activate and undergo phenotypic conversion to myofibroblasts, altering their function. Persistence of myofibroblasts may lead to a failure to resolve repair processes such as wound healing, which in turn can lead to fibrosis and replacement of functional tissue with non-functional scar, can promote cellular transformation and metastasis in cancer, and can contribute to tissue aging. The negative impacts of fibroblast activation on tissue function can range from being relatively minor or cosmetic, to life-threatening.

Fibroblasts are flat, spindle-shaped cells that are easily distinguishable from other cell types. As supportive stromal cells, they help to maintain tissue homeostasis, with roles that range from generating extracellular matrix (ECM) proteins, to producing or degrading growth factors and cytokines in inflammatory processes. Once activated, fibroblasts are key players in certain physiological and pathophysiological conditions such as in wound healing, fibrosis, and tissue development and remodeling (Figure 1). Myofibroblasts are larger in size, with a highly active endoplasmic reticulum to support their synthetic character, and exhibit contractile characteristics similar to smooth muscle cells as they form stress fibers that are frequently comprised of alpha-smooth muscle actin (α-SMA) [1].

Fibroblasts exhibit tissue-specific properties both in normal homeostasis and following injury. They produce and secrete all components of the ECM, including structural proteins such as collagen and elastin, the expression of which varies among tissues based on the required tensile strength or elastic distensibility. The specific collagens expressed also vary significantly across tissues, with some forming fibers while others contribute to reticular networks. In addition, fibroblasts and myofibroblasts secrete adhesive proteins, and a space-filling ground substance including glycosaminoglycans and proteoglycans that provide tissue support and resistance [2].

While fibroblasts are heterogeneous not only across tissues, but also within individual tissue types, they tend to fulfill similar supportive functions. Conversely, this variety makes identification through the use of specific marker proteins challenging. Many proteins that are be highly expressed in fibroblasts may also be expressed in other cell types, such as pericytes or smooth muscle cells. Thus, a panel of proteins may be used to denote fibroblasts, although this selection can vary across tissues, including individual collagens, desmin and other ECM proteins. Given their mesenchymal origin, fibroblasts may be identified through positive expression of mesenchymal markers such as vimentin, although this, too, is expressed by macrophages and neurons. Similarly, the collagen receptor Discoidin Domain Receptor 2 (DDR2) has been used to mark cardiac fibroblasts, but is also found on leukocytes and tumor cells [3].

Upon activation, fibroblasts change their phenotype to progressively become myofibroblasts, and alter their gene expression patterns. Similar to fibroblasts, it remains challenging to identify myofibroblasts solely on the basis of a specific marker, thus a collection of markers may be used. Up-regulation of α-SMA, the ED-A splice variant of fibronectin (FN), and smooth muscle myosin heavy chain (SMemb) have been used to varying degrees, and collagen expression may also increase significantly [4,5]. Periostin is expressed highly in cardiac myofibroblasts but not fibroblasts, and marks human cardiac fibrosis [6].

There is also heterogeneity as fibroblasts transition to myofibroblasts. The term “proto-myofibroblast” was previously coined to denote an intermediate state, in which α-SMA may be expressed but is not incorporated into stress fibers [7]. More recent work using single cell RNA sequencing coupled with cluster analysis of gene expression has indicated that tissues may host a variety of populations of cells ranging from fibroblasts to myofibroblasts, and these populations are altered in disease [3,8]. Thus, the terms “fibroblast” and “myofibroblast,” while useful conceptually, fail to capture the true complexity of the cell populations that comprise these roles [1,9].

### 1.2. Role of Fibroblasts in Homeostasis and Injury

#### 1.2.1. Cardiac Fibroblasts

In the heart, resident fibroblasts contribute to tissue integrity in normal physiological conditions. They create an interconnected meshwork of protein fibers that surround the myocytes, secreting a heart-specific structural ECM that is dense, irregular, and composed primarily of collagens, proteoglycans, and glycoproteins to support cardiac contractility [10]. In addition, significant evidence has linked cardiac fibroblast function to the electrophysiological conduction properties of the heart through cardiomyocyte electrical coupling, vascular maintenance, and stress sensing [11].

In response to environmental alterations, including physical and chemical changes, fibroblasts undergo a change of phenotype that alters their morphological and behavioral characteristics, in settings ranging from development to disease. These changes are tissue-specific, depending on the specific nature of mechanical stresses and/or chemical modulators such as cytokines and growth factors. For example, in cardiac muscle following myocardial infarction (MI), cardiomyocyte necrosis attracts reparative fibroblasts to the area of injury via release of damage signals and tissue invasion of secretory inflammatory cells. Release of signaling molecules such as Transforming Growth Factor β (TGFβ) and Tumor Necrosis Factor (TNF) activate resident cardiac fibroblasts. Increased mechanical stress on the cardiac wall further stimulates resident fibroblasts adjacent to the site of injury to become activated and infiltrate the injured region. These motile, activated fibroblasts contribute to the initial deposition of a reparative matrix including ED-A FN and non-fibrillar collagens that serves to reduce risk of aneurysm at the site of damage. This immature matrix accumulation promotes further activation of fibroblasts to myofibroblasts and promotes chemotactic responses to cytokines and growth factors.Myofibroblasts then go on to generate mature ECM characterized by fibrillar collagens such as type I and III, forming the cardiac infarct scar. The scar tissue matures, with an increase in ECM cross-linking and eventual induction of apoptosis of most (but not all) reparative myofibroblasts. Fibroblast plasticity thus plays a crucial role in preserving myocardial integrity from cardiac rupture through this wound healing process [12].

Resident cardiac fibroblasts represent the major source of myofibroblasts in cardiac repair. Fibroblasts infiltrating from blood-derived progenitors, originating from pericytes, or from the endothelium through endothelial-to-mesenchymal transition have previously been reported to also contribute to cardiac myofibroblasts [13,14,15]. However, recent lineage tracing work has demonstrated that nearly all myofibroblasts post-infarction and in pressure overload arise from resident fibroblasts [16,17,18]. Myofibroblasts are the primary cells responsible for matrix synthesis in the injured heart. In other tissue types, myofibroblasts arise from a broader variety of sources than in the heart, such as from hepatic stellate cells in the liver.

#### 1.2.2. Dermal Fibroblasts and Wound Healing

In the skin, dermal fibroblasts are present within the dermis layer, and they are the main cells that facilitate skin repair and recovery by generating connective tissue proteins when injury occurs. Different dermal layers can possess diverse subpopulations of fibroblasts that can be morphologically and functionally unique [19]. Different levels of expression of collagens, with variable ratios of collagen type I and III, occur depending on their depth within the dermis. Superficially located fibroblasts tend to produce higher levels of collagenases [20].

When a wound occurs, paracrine factors from macrophages induce phenotype conversion of dermal fibroblasts. Differentiated myofibroblasts tend to contract due to the presence of stress fibers, as well as cell–cell and cell–matrix connections that occur via gap junctions and desmosomes. Collagen I and III fibrils are anchored to the cells to help contract the wound and promote closure, promoting faster wound healing [21,22]. A recent study employed cell fate-mapping approaches to demonstrate that most of the reparative fibroblasts arise from mesenchymal progenitors which acquire the functional plasticity to mediate wound regeneration in the neodermis and scar formation in the periphery [23].

#### 1.2.3. Cancer-Associated Fibroblasts

Other studies have investigated the role of fibroblast cellular plasticity in tumor progression and metastasis. In cancerous tissue, fibroblasts are the key players in all stages of disease initiation, progression, and therapy resistance, including metastasis and up to tissue damage. Cancer-associated fibroblasts (CAFs) up-regulate ECM components, and demonstrate increased capacity to proliferate and migrate. CAFs also release several growth factors and cytokines to promote inflammation, promote tumor progression through secretion of matrix metalloproteinases (MMPs) to degrade ECM, and induce the release of vascular endothelial growth factor A (VEGF-A) to promote the formation of new blood vessels, thus facilitating nutrient access to drive tumor growth and expansion [24].

A recent fibroblast proteome profiling study of tumor samples from human skin, lung, and bone marrow reported that tumor-associated fibroblasts exhibit protein expression profiles that are tissue-specific. In contrast, treating non-tumorous fibroblasts from the same tissues with pro-inflammatory interleukin IL-1beta resulted in both common and unique changes in inflammation-related protein expression, suggesting that the tumor microenvironment is important in regulating fibroblast behavior [25].

Studies on multicellular breast cancer clusters have revealed that the origin of invasive and metastatic CAFs is through the induction of epithelial–mesenchymal transition (EMT). Tumor clusters, which are frequently present in breast cancer tumor stroma, can be composed of either highly epithelial cells or a hybrid of both epithelial/mesenchymal cancerous cell populations. Both of these cell populations exhibit distinctive oncogenic cell–cell adhesion molecules, such as carcinoembryonic antigen-related cell adhesion molecule 5 (CEACAM5) and CEACAM6, which could result in increased tumor cell cluster formation and metastasis [26].

Similarly, in the setting of melanoma, stromal fibroblast populations of the tumor microenvironment including normal fibroblasts and melanoma-associated fibroblasts (MAFs) influence melanoma growth and health outcomes. Resident dermal fibroblasts are the main source of CAFs in melanoma, and have been found to highly express α-SMA, among other markers of fibroblast activation and differentiation including fibroblast activation protein (FAP), fibroblast-specific protein 1 (FSP-1), osteonectin, desmin, platelet-derived growth factor receptor (PDGFR), periostin, neuron-glial antigen-2 (NG2), and vimentin [27]. In vitro co-culture experiments have shown bidirectional influence altering gene expression in both fibroblasts and melanoma cells. Co-cultured fibroblasts were shown to induce genes associated with matrix degradation, such as MMP-1 and MMP-3, as well as cell proliferation and proinflammatory pathways, including IL-1β, IL-8, and CCL2 by paracrine and cell–cell interactions between melanoma cells and normal dermal fibroblasts, thus gradually acquiring a cancer-promoting growth response [28].

#### 1.2.4. Fibroblasts and Aging

Fibroblast heterogeneity can impact the aging process in different tissues. For example, in vitro studies using young and aged human skin samples have revealed that gene expression levels in the dermal fibroblast are significantly altered with age, while the cell growth rate was reduced. In particular, it was found that *COL1A1*, *COL4A1*, and *COL7A1* gene expression was decreased in human dermal fibroblasts from elderly donors compared to young ones [29]. This result is suggestive of reduced skin rejuvenation in the elderly. Additional work has explored the correlation between aging and stiffening of dermal fibroblasts. The viscoelastic properties of fibroblasts from older donors exhibited ~60% more rigidity compared to cells from younger donors [30].

While the fibroblast-to-myofibroblast transition has not been fully investigated in the context of the aging process, it was reported that age-related impairment in wound healing is correlated with impaired fibroblast to myofibroblast activation through hyaluronan reduction. As hyaluronan is an important connective tissue glycosaminoglycan, with roles in maintaining ECM stability via cell–cell adhesion and in wound healing, this study suggests that hyaluronan loss with increasing age impairs the wound healing process [31].

#### 1.2.5. Fibroblasts and Tissue Fibrosis

The importance of fibroblast plasticity and heterogeneity extends beyond wound healing to pathological tissue fibrosis. Upon injury, the structural integrity of the tissue is lost, as mechanical stress activates fibroblasts and induces their activation into proto-myofibroblasts [32]. In the heart, persistent survival of myofibroblasts after MI can exert significant negative consequences on cardiac function and contractility. Cardiac fibrosis is a serious condition that may contribute to the development of cardiac arrythmias and heart failure [33].

A variety of transcription factors and intracellular signaling pathways are associated with cardiac fibroblast activation and scar formation in the heart. This includes the TGFβ/Smad signaling pathway—a potent inducer of wound healing, fibroblast activation, and scarring. TGFβ is both secreted from a wide variety of cells, and released from stores within the ECM in response to physical forces. Transmembrane integrins connect the external ECM with the internal actin cytoskeleton, transducing mechanical signals between the extracellular and intracellular compartments to contribute to tissue-specific physiological functions including cellular differentiation, migration, and proliferation [34]. Secreted TGFβ is stored in the ECM in a latent form via its interaction with latency-associated peptide (LAP) as part of a larger complex. Integrins αvβ3, αvβ5, αvβ6, and αvβ8 bind to the RGD sequence of LAP, and these interactions govern the sequestration of activation of TGFβ [35,36,37,38].

Following activation and release, TGFβ binds to its cognate cell surface receptors—heteromeric complexes comprised of subunits TGFBR1 and TGFBR2—resulting in activation of both Smad-mediated and non-Smad-mediated intracellular signaling pathways. In the Smad signaling pathway, receptor Smads (including Smad2 and Smad3) become phosphorylated, and in combination with Co-Smad4, translocate to the nucleus and transactivate a downstream pro-fibrotic gene program [39]. Inhibitory Smad7 serves as a negative feedback regulator this mechanism [40]. Smad7 interferes with the recruitment of Smad2/3 upon TGFβ activation by recruiting E3 ubiquitin ligases to target components of this pathway for degradation [41]. The non-Smad-mediated pathways downstream of TGFβ include a variety of kinases such as c-jun kinase and p38.

The transcription factor scleraxis has been identified as a master regulator of the fibrotic signaling pathway in the heart: it binds directly to pro-fibrotic gene promoters such as *COL1A2* to upregulate target gene expression, including various ECM proteins such as fibronectin and vimentin. Notably, scleraxis was sufficient to induce fibroblast to myofibroblast activation and conversion, was required for TGFβ-mediated fibroblast activation, and could also induce EMT via direct transactivation of *Twist1* and *Snai1* [42,43].

Fibrosis in the heart can arise from a wide variety of insults. For example, diabetic cardiomyopathy is marked by altered myocardial structure and function in diabetic patients without other related cardiac risk factors. Diabetes modifies metabolism in the heart via enhancement of fatty acid oxidation and attenuation of glucose oxidation, resulting in reduced energy production and increased susceptibility to ischemia/reperfusion injury, in turn increasing the risk of death due to heart failure [44]. Interstitial and perivascular fibrosis are the main histological features of diabetic cardiomyopathy, adversely impacting ventricular compliance and electrical properties [45].

Kidney function is adversely affected by renal fibrosis. Myofibroblasts are derived from a variety of cells within the kidney, such as vascular smooth muscle, mesangial, resident stem, tubular epithelial, vascular endothelial cells and pericytes [46]. In end-stage renal disease, each of these cell types may contribute to the fibrotic process. Various pro-fibrotic agents (such as angiotensin II (AngII), endothelin and TGFβ) are upregulated in chronic kidney disease, altering renal morphological and functional capacity through shifting protein gene expression towards the fibrotic state. An important player in this process is the renin–angiotensin system, which induces the expression of ECM proteins via activation of members of the TGFβ/bone morphogenetic protein (BMP) superfamily [47].

Post-injury scarring is an extreme example of tissue fibrosis. In the skin, it is marked by excessive proliferation of fibroblasts, along with massive deposition of ECM (primarily collagen), reduced tissue tensile strength and elasticity, and the loss of hair follicles. Human dermal wounds subjected to elevated tension often result in extensive scarring compared to those experiencing minimal tension. The more stretch that is applied, the more tension is generated on dermal fibroblasts, which induces chemical signals to drive fibrosis [48]. For example, activation of focal adhesion kinase in fibroblasts results in increased scar formation. This kinase in turn acts through extracellular-related kinase to directly trigger secretion of monocyte chemoattractant protein-1, a chemokine linked to human fibrotic disorders [49].

Cell–cell adhesion between dermal fibroblasts has also been implicated in the progression of scar formation. It was found that the expression of cell adhesion molecules such as cadherin-11 (CDH11) on dermal fibroblasts is elevated in the skin of patients suffering from systemic sclerosis or scleroderma [50].

### 1.3. Fibroblast Metabolism

The decision point for fibroblasts to institute wound repair or induce scarring is influenced by the microenvironmental stresses and the fibrotic signaling pathways acting upon fibroblasts. In either situation, the significant synthetic capacity of fibroblasts comes with elevated metabolic needs. New work is providing insight into the metabolism of fibroblasts and myofibroblasts, and how alterations in metabolism may contribute to their fibrotic potential. A study of primary human dermal fibroblasts showed that quiescent fibroblasts are metabolically active, as they divert glucose to the pentose phosphate pathway to generate NADPH for nucleotide biosynthesis, free radical detoxification, and energy generation. These fibroblasts degrade and resynthesize proteins and fatty acids, and secrete large amounts of protein into the ECM to maintain homeostasis [51,52].

Additional studies have investigated the role of cellular metabolism specifically in the activation of fibroblasts and their conversion to myofibroblasts, with subsequent links to fibrosis and tumorigenesis [53]. The major metabolic pathway in fibroblasts is typically glycolysis. This pathway regulates the metabolism of glucose through a series of enzymatic activities to produce pyruvate and ATP. Conversely, defects in fatty acid metabolism can induce the profibrotic phenotype of alveolar epithelial cells and macrophages, and can activate fibroblasts to myofibroblasts in the setting of mouse lungs subjected to experimental pulmonary fibrosis, a model of idiopathic pulmonary fibrosis [54]. Glutaminolysis is another metabolic pathway important for energy production in highly metabolic fibroblasts during their activation. Glutaminolysis converts glutamine to glutamate and ammonia by the rate-limiting enzyme glutaminase (GLS). Several studies have investigated the role of glutaminolysis in hepatic and pulmonary fibrosis, revealing the importance of this mechanism as a fuel source to drive fibrosis [55,56]. A recent study by our lab has revealed that scleraxis upregulates GLS1 expression to promote glutaminolysis in cardiac fibroblast activation and conversion to myofibroblasts, suggesting a similar metabolic pathway to support fibrosis exists in the heart as in the liver and lung [57].

Understanding fibroblast biology and activation to myofibroblasts is thus key to understanding processes such as wound healing, tumor progression, fibrosis, and scarring, as well as the potential development of relevant therapies. A number of investigators in Canada have made notable contributions to the field, and in the next section, we broadly examine some of these discoveries. Please note that, due to space limitations, this list is not exhaustive, and exclusions or oversights are not intentional; for similar reasons, it also does not reflect a number of rich collaborations between various Canadian laboratories.

## 2. Canadian Contributions to Understanding Fibroblast Biology

### 2.1. TGFβ Signaling Pathway Regulators

As noted above, the TGFβ signaling pathway is known to play a central role in the activation of fibroblasts to myofibroblasts and subsequent fibrosis. Important insights into how TGFβ signaling itself is modulated have come from the lab of Boris Hinz. They have shown that integrins αvβ5 and αvβ3, which bind to latent TGFβ, are increased during the fibroblast-to-myofibroblast transition both in vitro and in vivo, and that the latent TGFβ in cardiac fibroblasts was activated by these integrins, leading to fibroblast activation. These integrins were able to compensate for each other’s function, and blocking the integrins significantly reduced fibroblast activation, showing promise as a novel anti-fibrotic strategy [58].

The laboratory of Christopher McCulloch has studied the role of α11 integrin in diabetes-induced fibroblast activation and conversion to myofibroblasts. They reported that α11 expression is much higher in cardiac fibroblasts isolated from diabetic rats, and in human cardiac fibroblasts plated on glycated collagen, compared to controls [59,60]. They showed that glycated collagen triggered the TGFβ signaling pathway, which in turn enhanced α11 expression through Smad 2/3 binding to its gene promoter. They also found that increased α11 expression stimulated collagen synthesis, activated TGFβ_2_ and resulted in fibroblast to myofibroblast activation. Thus, blocking this TGFβ_2_-integin signaling pathway may reduce fibrosis in diabetic cardiomyopathy.

The Hinz laboratory has also reported that, in response to mechanical stress, the expression level of both ED-A FN, the predominant splice variant of fibronectin in fibrosis, and latent TGFβ binding protein-1 (LTBP-1) was increased, with both proteins colocalized in the ECM. The ED-A domain of fibronectin enhanced binding of LTBP-1 to fibronectin, in turn leading to LTBP-1 incorporation into the fibroblast ECM. Blocking this interaction, using either competitive domain peptides or specific antibodies, could be an effective strategy to prevent TGFβ activation and in turn block fibrosis [61]. The Hinz lab has further reported that adhesion protein CDH11 is present at the contact region of macrophages and myofibroblasts, and serves to activate latent TGFβ produced by the macrophages and supplied to the myofibroblasts. Inhibiting CDH11 could provide a means to attenuate the interaction of macrophages and myofibroblasts to also impact fibrosis [62]. They also found that the focal adhesion protein kindlin-2 was upregulated in activated cardiac fibroblasts in vitro and in vivo, and enhanced fibroblast to myofibroblast transition by regulating α-SMA at the promoter level, making it a prime target for anti-fibrotic strategies [63]. Collectively, these findings have provided new insight into the importance of cell–cell and cell–matrix interactions, and revealed the potential therapeutic importance of targeting these interactions.

Endogenous negative regulators of TGFβ signaling are of particular therapeutic interest given their potential roles in attenuating or blocking pro-fibrosis signals. Ian Dixon’s lab previously investigated the localization of inhibitory Smad7, which counteracts the receptor Smads 2 and 3, in cardiac fibroblasts. They showed that, upon activation of TGFβ signaling, there was a transient increase in the level of Smad7, suggesting the presence of a negative feedback loop [64]. In contrast, Smad7 expression was significantly decreased in both viable and scar tissue post-MI. Overexpression of Smad7 supressed expression of fibrillar collagens I and III. Decreased expression of inhibitory Smad7 may thus contribute to cardiac fibrosis after MI, suggesting its potential for anti-fibrotic strategies. More recently, the Dixon lab has explored the role of the proto-oncoprotein c-Ski (SKI), an inhibitor of TGFβ, in cardiac fibroblast activation. The 95 kDa isoform of SKI impaired the function of phosphorylated Smad2 and inhibited pro-collagen I synthesis, as well as myofibroblast contractility both basally and upon induction by TGFβ. SKI had the net effect of attenuating the myofibroblast phenotype, and reduced myofibroblast viability by inducing apoptosis and inhibiting autophagy. New strategies to increase expression or function of SKI may thus be beneficial in the context of matrix remodelling and cardiac fibrosis [65,66,67]. The laboratory of Kim Connelly recently reported an alternative approach to modulate Smad2/3 signaling. SRT1720, a pharmacological activator of sirtuin 1, attenuated remodeling and fibrosis in a mouse pressure overload model [68]. SRT1720 attenuated Smad2/3 phosphorylation and acetylation in isolated cell studies, resulting in the reduced expression of type I collagen and α-SMA. Thus, attenuating TGFβ/Smad signaling has promise for improving cardiac function in pressure overload.

CD109 is a TGFβ co-receptor that negatively regulates TGFβ signaling. The laboratory of Anie Philip has reported that CD109 expression is elevated in skin samples from patients with systemic sclerosis (SSc)/scleroderma, as well as in skin fibroblasts cultured from SSc patients [69]. Knockdown of CD109 in skin fibroblasts from non-SSc and SSc individuals resulted in evidence of TGFβ signaling activation, including Smad2/3 phosphorylation and increased ECM protein expression, whereas CD109 gain-of-function reduced ECM protein expression. In a separate study, CD109 over-expression in a mouse bleomycin-induced scleroderma model led to histological evidence of reduced skin fibrosis and a decrease in Smad2/3 phosphorylation [70]. The team hypothesized that the elevation of CD109 in SSc patients may thus represent an adaptation to elevated TGFβ signaling, with potential value to reduce fibrotic disease as supported by the in vivo mouse scleroderma studies.

While increased ECM synthesis is most often associated with fibrosis, remodeling of the matrix via alterations in collagen crosslinking or degradation of matrix components by matrix metalloproteinases is also common. Attempts to alter matrix remodeling, either alone or in conjunction with efforts to improve the function of damaged tissue, have met with some success in addressing the challenges of tissue fibrosis. Paul Fedak’s team engrafted vascular smooth muscle cells in an induced MI rat model and demonstrated that cell transplantation prevented maladaptive ECM remodelling post-MI, improved cardiac function, attenuated myofibroblast activation, and increased the expression of basic fibroblast growth factor/FGF2 (inhibitor of TGFβ-induced fibroblast activation) and tissue inhibitor of matrix metalloproteinase (TIMP) 2, an endogenous inhibitor of MMP2 [71]. His lab further explored the dual nature of TIMP2, showing that low levels promoted fibroblast activation and maintained ECM homeostasis, while high levels had inhibitory effects on activation [72].

Zamaneh Kassiri and Gavin Oudit have extensively collaborated on the study of TIMPs as modulators of fibrosis, and have explored the complex functions of TIMPs in contributing to cardiac pathology beyond their basic function of inhibiting MMPs. They showed that different TIMPs impact myocardial remodelling in different ways in response to AngII: while TIMP2 deficiency increased hypertrophy and impaired active ventricular relaxation, knockout of TIMP3 induced cardiac fibrosis and increased passive ventricular stiffness. Using cardiac fibroblasts isolated from TIMP3 knockout mice treated with AngII for 2 weeks, they reported that the complexity of cellular events mediating myocardial fibrosis was post-translational matrix protein regulation rather than via de novo synthesis of collagen [73]. More recently, their labs have explored the role of TIMP1, and have shown that, similar to TIMP2 and TIMP3, it plays roles independent of its MMP inhibitory function in cardiac fibroblasts through interactions with CD63 and integrin β1, leading to activation and nuclear translocation of Smad2/3 and β-catenin, which in turn induce de novo synthesis of collagen I and III [74]. Targeting TIMP1 may thus provide a novel avenue for the development of anti-fibrosis strategies. In other studies, these labs have explored the protective effect of a disintegrin and metalloproteinases (ADAMs), specifically ADAM15 and ADAM17, following MI. While ADAM17 promoted angiogenesis through VEGFR induction, ADAM15 was required for optimum collagen cross linking and scar formation to prevent left ventricular rupture in the infarcted heart [75,76].

Carolyne Baglole, working with David Eidelman and Ilan Azuelos, has reported that TGFβ-induced lung fibroblast activation to myofibroblasts is accompanied by nuclear export of Human antigen R (HuR), a protein that stabilizes mRNAs, and showed that knockdown of HuR attenuated TGFβ-induced expression of α-SMA, fibrillar collagens and fibronectin [77]. HuR appears to function via direct binding to mRNAs for *COL1A1*, *COL3A1*, *ACTA2*, and *FN*, and it was found that HuR is localized to a greater degree in the cytosol in cells from mouse lung fibrosis models as well as human idiopathic pulmonary fibrosis patients. HuR may thus promote fibrosis by stabilizing mRNAs encoding fibrosis/ECM genes.

### 2.2. Growth Factors and Cytokines

While TGFβ is arguably the most potent chemical activator of fibrosis, various other growth factors and cytokines have also been demonstrated to play important roles in promoting or inhibiting fibrosis. Ian Dixon’s laboratory has studied a member of the IL-6 family of cytokines, cardiotrophin-1 (CT-1), which is increased in the serum of patients suffering from ischemic heart disease. They have shown that CT-1 induced cardiac fibroblast proliferation, migration, and protein synthesis via mechanisms involving the Jak/STAT, MAPK and PI3K/Akt pathways, and furthermore increased the synthesis of a marker of mature collagen, procollagen-1-carboxypropeptide (PICP), implicating CT-1 as a modulator of fibroblast function and infarct scar formation and remodelling post-MI [78,79]. The Kassiri and Oudit laboratories have reported on the role of TNF, an inflammatory cytokine associated with various health problems including cardiomyopathy, cardiac hypertrophy, aortic stenosis, and mitral valve regurgitation, and have showed how it variably impacts MMPs in cardiomyocytes as compared to cardiac fibroblasts. In response to pressure overload and in comparison to wild-type mice, TNF knockout mice exhibited reduced collagenase activity, lower superoxide levels and PI3K activity, attenuated cardiomyopathy, and reduced interstitial and perivascular fibrosis as well as cardiac remodeling [80].

The laboratory of Andrew Leask has focused on CAFs and their crosstalk with other cells in the tumor microenvironment. Their work has shown that CCN2 (formerly known as Connective Tissue Growth Factor or CTGF) expressed by CAFs is essential for tumor vascularization, making it a novel target for treating melanoma [81]. Extending that work, his lab has shown how members of the CCN family of matricellular proteins contribute to tissue fibrosis. CCN1 secreted from fibroblasts plays a major contributory role in skin fibrosis via the regulation of collagen synthesis, while CCN2 regulates myofibroblast activation, making both molecules intriguing anti-fibrotic targets [82]. His recent work has focused on repurposing verteporfin, a drug used in the treatment of macular degeneration, to inhibit the transcription co-activator YAP, which itself plays a major role in regulating expression levels of CCN1 and CCN2, as a novel approach to treating fibrosis [83]. Recent work from the Dixon laboratory has examined YAP/TAZ signaling in the Hippo pathway in cardiac fibroblast activation, and demonstrates that SKI induces proteasomal degradation of TAZ to inhibit the myofibroblast phenotype [84].

Alterations in cardiac ECM structure and abundance can alter the electrical properties of the heart, contributing to arrhythmogenesis. The laboratory of Robert Rose has been examining the role of the cardioprotective natriuretic peptides, and has reported that mice lacking natriuretic peptide receptor C (NPR-C) show exaggerated fibrosis in response to AngII administration compared to those treated with AngII alone [85]. Conversely, the NPR-C agonist cANF attenuated fibrosis induced by AngII, resulting in reduced induction of atrial fibrillation. Similar effects were noted in aged mice lacking NPR-C, suggesting a general protective effect of this receptor that reduces adverse ECM and electrical remodeling [86].

### 2.3. Hypoxia

The Hinz lab has reported that hypoxia induces proliferation and reduces apoptosis of subcutaneous fibroblasts (SCFs), and at the same time reduces myofibroblast contraction and activation while rendering the cells desensitized to TGFβ [87]. However, this effect was reversible, as switching to normal 21% O_2_ or applying mechanical stress restored myofibroblast activation. Notably, different fibroblasts responded differently to hypoxia. While cardiac fibroblasts behaved similarly to SCFs, lung and liver fibroblasts showed the opposite effect: increased myofibroblast activation in response to hypoxia. It was noted in the Hinz paper that earlier work by Roy et al. had suggested that cardiac fibroblasts exposed to hypoxic conditions appear to perceive ambient oxygen level as a reference point, such that even a small increase in oxygen concentration put the cells into hyperoxia, in turn resulting in activation of fibroblasts to myofibroblasts [88]. Thus, hypoxia in the heart may itself not be pro-fibrotic—rather, the danger may occur during reoxygenation, paradoxically promoting remodeling and fibrosis, although the contribution of concomitant inflammation cannot be discounted. This observation may also help to explain the important differences in fibrosis presentation in ischemic vs. non-ischemic cardiac patients, which has significant impact on outcomes: a presentation of major non-ischemic fibrosis resulted in a hazard ratio for event-free survival of 2.3 relative to ischemic fibrosis/myocardial infarction [89]. In contrast, the lungs and liver would appear poised to respond to hypoxia by induction of fibrosis. These results emphasize the heterogeneous nature of fibroblasts and their environmental responses, and the need to compare and contrast fibroblasts from different tissue types.

### 2.4. Transcription Factors

Just as the intracellular signaling pathways governing fibroblast activation and fibrosis are being elucidated, a growing number of transcriptional regulators, downstream of these pathways, have begun to be identified and explored as potential therapeutic targets. Our lab has focused on a basic helix-loop-helix transcription factor called scleraxis, and which is primarily known as a regulator of cartilage and tendon formation and repair. The composition of the cardiac ECM is surprisingly similar to that of tendons, being composed primarily of type I collagen, even if its organization is very different; thus, we originally reasoned that scleraxis may govern the expression of fibrillar collagens in the heart. We have found that scleraxis directly transactivates the promoters of a variety of genes involved in ECM production and fibrosis, including *Col1α2*, fibronectin, vimentin, *MMP2*, and α-SMA, and does so downstream of TGFβ via both canonical Smad and non-canonical signaling pathways [42,43,57,90,91,92,93,94,95]. Scleraxis alone is sufficient to activate fibroblasts to become myofibroblasts, inducing focal adhesion and stress fiber formation and inhibiting cell proliferation and migration, and knockout or knockdown of scleraxis in cardiac fibroblasts completely attenuates TGFβ- or stretch-mediated activation of these cells. Scleraxis is highly expressed in the post-MI scar [90]. We have reported that scleraxis physically interacts with Smad3, and is necessary for the recruitment of transcriptional complexes to the *Col1α2* promoter in response to TGFβ. Scleraxis thus seems to be critical in linking pro-fibrotic signals to fibrotic gene expression.

Scleraxis is also important in the development of cardiac fibroblasts prior to birth. Knockout of scleraxis resulted in a 50% decrease in the number of cardiac fibroblasts, with a loss of approximately one third of the basal ECM [42]. We hypothesized at the time that scleraxis may function in the mechanism by which epithelial cells in the heart transition to mesenchymal fibroblasts, and noted an increase in the expression of epithelial cell markers concomitant with a loss of mesenchymal markers in scleraxis knockout hearts. We subsequently demonstrated that scleraxis is sufficient to induce EMT through the direct transactivation of the *Twist1* and *Snai1* gene promoters, and loss of scleraxis attenuated the ability of TGFβ to induce EMT [43]. We have collaborated with the Dixon laboratory to examine the potential interplay between anti-fibrotic SKI and pro-fibrotic scleraxis, and found that scleraxis expression was inhibited by SKI [95].

Our current working model is that scleraxis is not specifically a driver of fibrosis, but rather an inducer of wound repair, one aspect of which may be fibrosis and scarring when the injury is unresolved. In this model, scleraxis functions to enable activation of fibroblasts to reparative myofibroblasts, effectively changing the phenotype of these cells by controlling the expression of a host of myofibroblast-associated genes as noted above. Indeed, as indicated above, we have shown that scleraxis regulates the expression of GLS1, the rate-limiting enzyme of the glutaminolysis pathway for energy generation to support the energy-intensive process of macromolecule synthesis in fibrosis [57]. Our activities are now focused on targeting scleraxis to attenuate fibroblast activation and in turn, ameliorate fibrosis and the resulting loss of cardiac function. This may involve direct drug targeting, however kinase inhibitors may also be useful in this regard, as we have shown that scleraxis is constitutively phosphorylated on two highly-conserved serine residues, and this phosphorylation is necessary for its function as a transcription factor [96,97].

The laboratory of Jeffrey Wigle has examined the emerging role of the zinc finger E box-binding homeobox 2 (Zeb2) transcription factor in regulating fibroblast activation as noted via increased expression of myofibroblast markers, reduced migration and increased contractile properties. Interestingly, Zeb2 deletion did not alter the expression of myofibroblast markers, however Zeb2 overexpression in fibroblasts caused activation to myofibroblasts, suggesting that Zeb2 is sufficient but not necessary for this process [98]. A collaboration between the Dixon and Wigle labs explored the link between SKI, Zeb2 and Meox2, an inhibitor of TGFβ-mediated EMT. Meox2 expression was reduced in the post-MI scar, and its overexpression inhibited fibroblast to myofibroblast activation, a function very similar to that found for SKI. They found that, while overexpression of SKI increased Meox2 expression, Zeb2 expression was attenuated, with similar results found in post-MI tissue samples. These results identified a novel signalling axis contributing to fibroblast to myofibroblast conversion involving these three proteins, revealing that SKI, through repression of Zeb2, derepressed Meox2 to attenuate the myofibroblast phenotype [99].

### 2.5. Collagen Receptors

Research on collagen has typically focused on its nature as a structural component of ECM, and on its overexpression in fibrosis. However, collagen also serves an important signaling role via its interaction with cells, providing information on matrix integrity and physical forces acting upon tissues. Michelle Bendeck’s laboratory has examined DDRs, which are novel tyrosine kinase collagen receptors that, upon binding to collagen, induce the up-regulation of MMPs. DDR1 is expressed both during embryonic development and in adult tissue, primarily in the skin, gut, brain, and kidney epithelium. DDR2 is present in the heart, skeletal muscle and connective tissue [100,101]. Dr. Bendeck’s team was the first to show the important role of DDR1 in lesion growth following vascular injury via its binding to collagen in the ECM, and also demonstrated its unique role in governing smooth muscle cell proliferation in response to injury [100,102]. Deletion of DDR1 in a mouse atherogenesis model reduced atherogenesis and increased evidence of vascular fibrosis, including increased expression of fibrillar collagens I and III, indicative of smaller and potentially more stable plaques [103].

### 2.6. Atrial Fibrillation and Atrial Fibrosis

Atrial fibrosis is common in patients suffering from atrial fibrillation, and can actually be contributory to the underlying arrhythmogenic substrate. The laboratory of Stanley Nattel has made significant inroads to understanding the interplay between fibrillation and fibrosis, including showing that simvastatin inhibited atrial structural remodelling in congestive heart failure in dogs, reducing atrial fibrillation incidence, atrial fibrosis, and conduction abnormalities [104]. Simvastatin similarly inhibited left ventricular remodelling, and notably prevented atrial fibroblast proliferation as well as activation of fibroblasts to myofibroblasts, noted as a significant attenuation of TGFβ-induced α-SMA expression.

To examine the role of atrial fibrillation in causing atrial remodeling and fibrosis, the Nattel laboratory examined the effect of medium from tachypaced atrial cardiomyocytes on fibroblasts. Atrial fibroblasts treated with culture medium from rapidly paced cardiomyocytes resulted in rapid activation of fibroblasts to myofibroblasts, with up-regulation of α-SMA, collagen and fibronectin, as well as reduced proliferation [105]. Tachypacing in dogs in vivo similarly up-regulated α-SMA, type I collagen and fibronectin; thus, atrial fibrillation and fibrosis appear to exhibit bidirectional regulatory effects upon one another, which may exacerbate disease pathogenesis.

The Nattel laboratory, working with Jean-Claude Tardif and Philippe Comtois, has also examined the role of microRNAs in the context of atrial fibrillation and fibrosis. Knockdown of miR-21 in a rat MI-induced heart failure model attenuated atrial fibrosis and greatly attenuated the duration of atrial fibrillation, with concomitant down-regulatin of miR-21 targets such as Sprouty-1 (an inhibitor of fibroblast proliferation) and fibrillar collagens [106]. Conversely, Nattel’s team has shown an anti-fibrotic role for miR-29b, which targets a number of extracellular matrix proteins, through gain- and loss-of-function approaches in isolated in canine atrial fibroblasts [107]. Knockdown of miR-29b in mice resulted in an increase of cardiac collagen content, and miR-29b expression is decreased in the serum of patients with atrial fibrillation and congestive heart failure, and in the atria of patients with chronic atrial fibrillation. The Nattel group has broadly characterized temporal miRNA expression in the failing tachypaced canine heart, finding that a number of fibrosis/ECM-related miRNAs are specifically altered in atrial fibroblasts [108]. Thus, cell-specific regulation of miRNA expression may play a key role in atrial remodeling and fibrosis, contributing to the induction or maintenance of atrial fibrillation substrate.

### 2.7. Matrix Stiffness

Physical forces and tissue mechanical stiffness are known to be important regulators of fibroblast cell biology, and changes in these forces (such as during cell stretch experiments) can be important triggers of fibroblast activation [94]. Controlling these factors in cell culture is important in interpreting experiments on primary isolated fibroblasts. Silicone elastomer, including polydimethylsiloxane (PDMS), is a frequently used material for producing stretchable cell culture substrates, however it is limited by high hydrophobicity which can contribute to low levels of cell attachment. The Hinz laboratory pioneered a novel, time-efficient and cost-effective method to covalently bind ECM proteins to PDMS, resulting in high coating density, enhanced fibroblast proliferation, higher fibroblast attachment, increased fibroblast spreading and prominent cell adhesion structures [109]. This approach enables in vitro cell culture experiments employing dynamic stretch conditions for studying mechanical signal transduction in various cell types, including fibroblasts, and other purposes such as functionalizing deformable body implants and tissue engineering.

The Hinz laboratory has also reported a novel approach for explanting and continuously passaging fibroblasts on silicone substrates having variable stiffness to mimic the various stages of lung health. They showed that increasing substrate stiffness increased myofibroblast proliferative capacity [110]. Explanting and passaging lung fibroblasts twice over 2 weeks on fibrotic stiff surfaces promoted cells to retain pro-fibrotic behaviours even when they were subsequently switched for 2 weeks to soft substrates mimicking a healthy environment. Similarly, lung fibroblasts maintained on soft, healthy-mimicking substrates showed delayed profibrotic responses upon subsequent culturing in on stiff, fibrotic substrate. Lung fibroblasts thus appear to have a mechanical memory following mechanical priming, conveying long-lasting effects on behaviour. A similar study in human dermal fibroblasts yielded similar results, suggesting that mechanical priming is a key feature of fibroblasts regardless of tissue source [111].

### 2.8. Inhibitors and Novel Anti-Fibrosis Strategies

Many laboratories are exploring means to inhibit or block the development or progression of fibrosis. The Fedak laboratory has explored the use of the calcium channel blocker tetrandrine, demonstrating that it can effectively attenuate human myofibroblast activation and in turn limit cardiac fibrosis, surprisingly via a mechanism not mediated by calcium channel blockade [112]. Similar to tetrandrine, his group has also demonstrated that the SGLT2 inhibitor empagliflozin converted myofibroblasts into a more quiescent phenotype, inhibiting ECM remodelling and attenuating pro-fibrotic marker expression [113]. His recent work with acellular bioactive scaffolds has shown that implanting them into the heart in a rat MI model, as well as in humans, can redirect cardiac fibroblasts from a fibrotic to a vasculogenic nature, resulting in improved cardiac function, inhibition of post-MI cardiac remodelling, and improving perfusion of the infarcted heart, providing a tractable clinical means to improve cardiac repair during surgical re-vascularization procedures [114].

Work from David Granville’s laboratory has implicated granzyme B (GzmB) in adverse cardiac remodeling and fibrosis. GzmB is elevated in human hearts with fibrosis, as well as in the mouse AngII hypertension model [115]. Knockout of GzmB reduced fibroblast number and fibrosis. Intriguingly, a topical inhibitor of GzmB, VTI-1002, was able to improve the healing of thermal skin wounds, with improved collagen organization, faster wound closure and increased strength of the healed wound [116]. Thus, GzmB may adversely impact remodeling in multiple scenarios where ECM organization is altered by injury or damage, and its blockade may be therapeutic. Whether GzmB inhibition may be effective specifically against tissue fibrosis is yet to be determined.

Collaborative work between Jeff Wigle and Thomas Netticadan has demonstrated that resveratrol can induce apoptotic death of cardiac fibroblasts and myofibroblasts, but not of cardiomyocytes [117]. Resveratrol also inhibited cardiac fibroblast and myofibroblast proliferation; thus, it may be effective as an anti-fibrotic and cardioprotective agent. In addition, recently, the Kassiri, Oudit, and McCulloch labs have explored a novel role for gelsolin in non-mechanical stress/AngII-induced cardiac fibrosis. Both in vitro and in vivo data in cardiac fibroblasts showed that gelsolin induces cardiac fibrosis by regulating the AMPK-mTOR-p70S6K pathway [118]. These novel regulators of fibroblasts and fibrosis show that there is clearly much more to be learned about this area.

The laboratory of Elissavet Kardami has extensively studied the role of different isoforms of FGF2 in cardiac pathology and repair. A low molecular weight FGF2 isoform (Lo-FGF2) has shown cardioprotective properties, while a high molecular weight isoform, Hi-FGF2, was associated with adverse effects. They reported that human atrial myofibroblasts secrete high levels of Hi-FGF2, and that Hi-FGF2 is readily detected at high levels in human pericardial fluid and atrial tissue extracts [119]. Notably, treating human atrial myofibroblasts with neutralizing antibodies against Hi-FGF2 attenuated the expression of collagen and α-SMA expression. These results suggest that targeting Hi-FGF2 may provide a novel means of reducing fibrosis.

Exciting new work from the laboratory of Fabio Rossi has described the presence of PDGFRα^+^/Sca-1^+^ cells dubbed “cardiac fibro/adipogenic progenitors”, and shown their importance in post-MI remodeling. In response to MI, these cells adopt a fibrogenic profile, but this can be pharmacologically blocked using tyrosine kinase inhibitors, resulting in improved cardiac function [120]. Conversely, activating these cells in otherwise healthy hearts led to fibrofatty infiltration. These results suggest that selectively altering the activation and fate of these cells could have therapeutic potential in a variety of settings associated with cardiac remodeling.

## 3. Conclusions

Tissue fibrosis remains stubbornly resistant to therapeutic intervention—despite many years of effort, only two medications have yet been approved for clinical use (both for idiopathic pulmonary fibrosis). This lack stems from a number of critical underlying issues that remain unresolved: a lack of specific targets that are effective in ameliorating fibrosis while avoiding off-target effects such as blunting normal wound healing or promoting oncogenesis; heterogeneity in fibroblasts both within tissues and across tissues, such that fundamental mechanisms of fibrosis may vary from tissue to tissue, or even on the basis of the specific pathophysiology in a given tissue; and finally, the ability to impact fibrosis with tissue specificity and temporal control.

Driven in part by this unmet need, fibroblast and fibrosis research has come to the forefront in the past decade, and new insights have accumulated more rapidly than ever before. New anti-fibrotic strategies seem likely to bear fruit, and significant efforts by many groups have provided important insights into diverging underlying mechanisms of fibrosis and fibroblast behavior between tissues. Canadian researchers have generated tremendous insight into fibroblast cell biology to date (Table 1)—and, as noted above, the work covered in this review is far from exhaustive. These researchers, and many others collaborating with them, will undoubtedly contribute significantly to the therapeutic management of fibrosis in the future.

## Figures and Tables

**Table 1:** Canadian contributions to understanding fibroblast biology and fibrosis. Effects of various regulators are divided into that those that promote fibrosis and/or scarring, and those that are inhibitory. Laboratory leaders and key collaborators are bracketed.

Regulator	Induces Fibrosis/Scar	Attenuates Fibrosis/Scar
TGFβ Signaling	Integrins αvβ5 and αvβ3 (Hinz)	Smad7 (Dixon)
	LTBP-1 and ED-A Fn (Hinz)	SKI (Dixon, Wigle, Czubryt)
	CDH11 (Hinz)	TIMP3 (Kassiri, Oudit)
	Low TIMP2 (Kassiri, Oudit)	High TIMP2 (Kassiri, Oudit)
	Kindlin-2 (Hinz)	CD109 (Philip)
	TIMP1 (Kassiri, Oudit)	SRT1720 (Connelly)
	ADAM15 (Kassiri, Oudit)	
	α11 integrin (McCulloch) HuR (Baglole, Eidelman, Azuelos)	
Other Growth Factors and Cytokines	CT-1 (Dixon)	Hi-FGF2 neutralizing antibodies(Kardami)
	TNF (Kassiri, Oudit)	NPR-C (Rose)
	CCN1/CCN2 (Leask)	
Hypoxia	Hypoxia in lung & liver (Hinz)	Hypoxia in skin and heart (Hinz)
Transcription Factors	Zeb2 (Wigle, Dixon)	Meox2 (Wigle, Dixon)
	Scleraxis (Czubryt, Dixon, Wigle)	
Collagen Receptors		DDR1 (Bendeck)
Other Regulatory Strategies	Gelsolin (Kassiri, Oudit, McCulloch)	Tetrandrine (Fedak)
	TAZ (Dixon)	Empagliflozin (Fedak)
	miR-21 (Nattel)	Resveratrol (Netticadan, Wigle)
		VSMC engraftment (Fedak)
		Acellular bioactive scaffolds (Fedak)
		Verteporfin (Leask) Simvastatin (Nattel) miR-29b (Nattel)

## Data Availability

Not applicable.
